# Progress in Medicine

**Published:** 1898-04-09

**Authors:** 


### Progress in Medicine.

### DISEASES OF DIGESTIVE ORGANS.

(*Concluded from page* 12.)

**Appendicitis.**—In a paper on the medical aspects of this affection Donald Hood[21] refers to important clinical types, the first where there is sudden perforation and peritonitis without previous symptoms of an important character, while the second shows localised inflammation in connection with the appendix or cæcum. The first is, of course, analogous to gastric ulcer or perforation in typhoid, and is very rare. The second may or may not present a tumour, and its symptoms include pain, a rise of temperature in all cases, tenderness on palpation, and even at an early stage some immobility of that part of the abdominal walls. As the disease advances, resistance, thickening, and tumour may appear, with frequently vomiting and diarrhœa or constipation. The writer acknowledges it is impossible to distinguish in the earlier stages between those cases where a coagulable lymph is poured out and those where pus is formed. Nor do hospital records afford information of much value as to the course and fatality of either class. Thus, out of 308 cases admitted into Guy's Hospital only 59 died, but many of them were admitted at an advanced stage, and little is to be learnt as to the efficiency of any line of treatment from them. Personally, he is in favour of the administration of opium at an early stage, and of avoiding all aperients. He has thus treated 35 patients without a death or abscess formation. Where relapses have occurred the same treatment has been followed with success. He insists upon absolute rest, slop diet, and half a grain of opium or more every six hours, preferably in the solid form.

Middleton,[22] in discussing the difficulties of diagnosis in abdominal disease, lays stress on the fact that pain is very misleading as a symptom. It is often referred to some distant part, and may be entirely absent even when the bowel is ruptured and the contents have escaped into the peritoneum. Then, again, the symptoms may point to a thoracic affection, though the trouble exists really in the abdomen, just as in some thoracic cases, such as basal pneumonia, the symptoms at first are those of peritonitis. In diseases of the abdomen, however, the doubt may not clear up, and the symptoms may point elsewhere through the entire course of the disease. Hence the importance of an examination of the rectum and of all other aids to localisation. A most remarkable instance of appendicular trouble is reported by T. A. McGraw.[23] The patient, a boy of seven years, began to have attacks of most violent abdominal pain, with choleraic diarrhœa and extreme tenesmus, followed by intervals of perfect health. There were no definite physical signs, and the attacks became more and more frequent. Operative interference showed that the affection was caused by the cæcum, which was of fœtal type, very long, and giving off the appendix from its extreme end. An intussusception had occurred, carrying the appendix and end of the cæcum downwards without, however, blocking up the ileo-cæcal opening, or causing any adhesions or obstruction. The condition seems to be almost unique, though intussusception of the ileum and cæcum into the colon are common. It is also rather difficult to see how such a process could be carried out, for the conditions are quite different from those present where an invagination in the continuity of the bowel is produced. The diagnosis of such cases must remain excessively difficult; the absence of digestive disturbances and the intermittent pain of intense character are important points, as well as the absence of any tendency to strangulation and obstruction. Other affections which might be mistaken for it are probably hernia of the linea alba, chronic partial obstruction of the bowels from flexions or stenosis, and chronic appendicitis. A volvulus of Meckel's diverticulum, reported by Carwardine,[24] may be mentioned here. Complete obstruction, vomiting, and distension occurred in an infant. A large cyst containing meconium was found, which communicated with the distended ileum by a minute canal and by an impervious cord with the lowest part of the small intestine. The whole mass had been twisted on itself, and peritonitis (with lymph and firm adhesions) had taken place even before birth. The cæcum and appendix were well formed.

**Hernia of the Linea Alba.**—A hernia in the epigastrum is rarely found, for it is generally small and difficult of detection, but its importance arises from the fact that it may give rise to symptoms of gastric disturbance. C. D. Aaron[25] reports an interesting case, and reviews the whole subject. The patient, aged 49, had for four years suffered from nausea and vomiting after food, with epigastric pain. The tongue was coated, there was headache, loss of flesh, and constipation. The position of the stomach was normal, and no disease could be found which was referable to it. Midway between the umbilicus and the end of the sternum was a lobulated mass of the size of a chestnut. An incision was made over the tumour, which was found to consist of fatty tissue, with a pedicle passing through an opening in the linea alba, and a peritoneal sac. After this hernia was removed and the aperture sutured the patient made a complete recovery, and all his symptoms disappeared permanently. Both Kuttner and Lindner have written on these herniæ, and pointed out the apparently intractable stomach symptoms which they may give rise to. They occur more often in men than in women, and vary in size from that of a bean to that of an egg. Sometimes they contain sub-peritoneal fat, and at others omentum, peritoneum, intestine, or even part of the stomach. The tumour is usually palpable, and very sensitive to pressure. Pain may be paroxysmal, and occur on bending, coughing, and particularly after taking solid food. Vomiting, nausea, flatulence, constipation, and neurasthenic symptoms may present themselves; and whether we have to do with a true hernia or with a sub-peritoneal lipoma, severe symptoms of this kind are an indication for operative interference.

As a method of diagnosis in obscure abdominal affections "blood counting" is showing itself of more and more importance. Herschell[26] dwells upon its value in cases where appendicitis is suspected. For the chief affections likely to be confounded with this disease are (*a*) colic from renal or biliary calculi or of a simple type, (*b*) obstruction from such causes as intussusception or volvulus, (*c*) ovarian neuralgia, (*d*) pyosalpinx, and (*e*)

typhoid. Now, in none of these is leucocytosis present except in pyosalpinx and the cholangitis and pyelitis, which may complicate respectively gall-stones or renal colic. On the other hand, it is present in appendicitis, except in the mildest and severest cases; that is, where the inflammation is too slight to produce the reaction, or the system is too poisoned to react. If the leucocytosis increases from day to day, it implies that the disease is spreading, or if it persists for several days, instead of gradually subsiding as the abscess becomes walled off, it indicates a large collection of pus. In both these cases operative treatment should not be delayed. Again, in jaundice, if we have leucocytosis the presumption is that we have to do with malignant disease, abscess, or cholangitis; and similarly in disease of the stomach, leucocytosis is present in many cases of malignant disease, acute gastritis, and slightly in hyperchlorydria, while it is absent in chronic gastritis, atonic dilatation, and ulcer, except after a hæmorrhage. The normal digestive leucocytosis is, however, affected differently by these diseases. Thus it is generally absent in cancer of the stomach, often in gastric catarrh, though present in ulcer and simple dilatation of the stomach. Capps[27] has reinvestigated the point, and found digestive leucocytosis absent in 15 out of 17 cases of cancer, and that it has about the same diagnostic value as the hydrochloric acid test in the same disease.

**Treatment.**—Thomas,[28] in speaking of tuberculous peritonitis, is opposed to laparotomy before other treatment has been thoroughly tried, especially since it has been shown that the operation only cures those cases where the tubercles are of the fibrous form, and that it is useless where they are caseous. He employed enemata of creasote in cod-liver oil in five cases with great success. Every night the patients received from 7 to 30 minims of creasote in 3 to 5 ounces of oil, and, if necessary, a few drops of laudanum were added to each injection. Mathieu[29] insists on the importance of relieving the constipation in membranous colitis, but avoids all drastic purgatives. Castor oil, cascara, saline purgatives, and copious injections are of value. The latter may be employed hot or with the addition of salicylate of soda for the relief of pain. Dignot found that injections of artificial serum, four to six drachms in amount, cause the disappearance of pain and false membranes entirely in a few weeks. If his results are confirmed, a remarkable advance in the treatment of a most intractable disease will have been made.

[21] Lancet, Sept. 18. [22] Glasgow Med. Jour., Nov. [23] B. M. Jour., Oct. 9. [24] B. M. Jour., Dec. 4. [25] Med. Rec., Nov. 20. [26] Clin. Jour., Jan. 12. [27] Practitioner, February. [28] B. Med. Jour., Jan. 22. [29] Amer. Jour. Med. Sc., Jan.

